# Assessment of prescribed vs. achieved fluid balance during continuous renal replacement therapy and mortality outcome

**DOI:** 10.1371/journal.pone.0272913

**Published:** 2022-08-25

**Authors:** Javier A. Neyra, Joshua Lambert, Victor Ortiz-Soriano, Daniel Cleland, Jon Colquitt, Paul Adams, Brittany D. Bissell, Lili Chan, Girish N. Nadkarni, Ashita Tolwani, Stuart L. Goldstein

**Affiliations:** 1 Division of Nephrology, Department of Internal Medicine, Bone and Mineral Metabolism, University of Kentucky, Lexington, Kentucky, United States of America; 2 Division of Nephrology, Department of Internal Medicine, University of Alabama at Birmingham, Birmingham, Alabama, United States of America; 3 College of Nursing, University of Cincinnati, Cincinnati, Ohio, United States of America; 4 Performance Analytics Center of Excellence, University of Kentucky, Lexington, Kentucky, United States of America; 5 Department of Pharmacy Practice and Science, University of Kentucky, Lexington, Kentucky, United States of America; 6 Division of Pulmonary, Department of Internal Medicine, Critical Care, and Sleep Medicine, University of Kentucky, Lexington, Kentucky, United States of America; 7 Division of Nephrology, Department of Medicine, Icahn School of Medicine at Mount Sinai New York, New York, NY, United States of America; 8 Charles Bronfman Institute of Personalized Medicine Hasso Plattner Institute of Digital Health Mount Sinai Clinical Intelligence Center, New York, NY, United States of America; 9 Center for Acute Care Nephrology, Cincinnati Children’s Hospital and Medical Center, University of Cincinnati, Cincinnati, Ohio, United States of America; Universita degli Studi di Perugia, ITALY

## Abstract

**Background:**

Fluid management during continuous renal replacement therapy (CRRT) requires accuracy in the prescription of desired patient fluid balance (FB_Goal_) and precision in the attainable patient fluid balance (FB_Achieved_). Herein, we examined the association of the gap between prescribed vs. achieved patient fluid balance during CRRT (%FB_Gap_) with hospital mortality in critically ill patients.

**Methods:**

Cohort study of critically ill adults with acute kidney injury (AKI) requiring CRRT and a prescription of negative fluid balance (mean patient fluid balance goal of negative ≥0.5 liters per day). Fluid management parameters included: 1) NUF (net ultrafiltration rate); 2) FB_Goal_; 3) FB_Achieved_; and 4) FB_*Gap*_ (*%* gap of fluid balance achieved vs. goal), all adjusted by patient’s weight (kg) and duration of CRRT (hours).

**Results:**

Data from 653 patients (median of 102.2 patient-hours of CRRT) were analyzed. Mean (SD) age was 56.7 (14.6) years and 61.9% were male. Hospital mortality rate was 64%. Despite FB_Goal_ was similar in patients who died vs. survived, survivors achieved greater negative fluid balance during CRRT than non-survivors: median FB_Achieved_ -0.25 [-0.52 to -0.05] vs. 0.06 [-0.26 to 0.62] ml/kg/h, *p*<0.001. Median NUF was lower in patients who died vs. survived: 1.06 [0.63–1.47] vs. 1.22 [0.82–1.69] ml/kg/h, *p*<0.001, and median %FB_Gap_ was higher in patients who died (112.8%, 61.5 to 165.7) vs. survived (64.2%, 30.5 to 91.8), *p*<0.001. In multivariable models, higher %FB_Gap_ was independently associated with increased risk of hospital mortality: aOR (95% CI) 1.01 (1.01–1.02), *p*<0.001. NUF was not associated with hospital mortality when adjusted by %FB_Gap_ and other clinical parameters: aOR 0.96 (0.72–1.28), *p* = 0.771.

**Conclusions:**

Higher %FB_Gap_ was independently associated with an increased risk of hospital mortality in critically ill adults with AKI on CRRT in whom clinicians prescribed negative fluid balance via CRRT. %FB_Gap_ represents a novel quality indicator of CRRT delivery that could assist with operationalizing fluid management interventions during CRRT.

## Introduction

Acute kidney injury (AKI) requiring renal replacement therapy (RRT) affects up to 10% of patients admitted to intensive care units (ICU) and is associated with increased morbidity and mortality [1–4]. RRT is commonly needed for management of electrolyte or acid-base derangements and fluid overload (FO) in patients with AKI in the ICU. Continuous renal replacement therapy (CRRT) is the most frequently utilized RRT modality to support critically ill patients with AKI and hemodynamic instability [5–7].

FO is well recognized as a contributor to mortality and multi-organ dysfunction during AKI and critical illness [8, 9]. Therefore, management of FO via net fluid removal rate (i.e., net ultrafiltration rate or NUF) is an important goal of CRRT. Higher FO at the time of CRRT initiation is associated with higher risk of 90-day major adverse kidney events, including mortality and decreased kidney recovery [10]. Further, FO at initiation of RRT has been associated with RRT-dependence at 1 year follow-up in AKI survivors [11]. In this context, FO represents a potentially modifiable risk factor that should be optimized and personalized through CRRT. The latter requires clinicians to carefully assess patient fluid balance status and target individualized fluid balance goals to manage FO. Unfortunately, fluid balance goals via NUF during CRRT are not always achieved due to patient inability to tolerate fluid removal due to hypotension or inadequate/interrupted CRRT delivery. Further, the same NUF may represent different patient fluid balance goals, which precludes the interpretation of the relationship of achieved NUF with mortality in the context of FO, as reported in recent observational studies [12–14].

Accordingly, the main objective of this study was to examine the association of the gap between prescribed vs. achieved patient fluid balance through NUF with hospital mortality in critically ill patients with AKI requiring CRRT. We hypothesized that the inability to achieve desired patient fluid balance goals increases the risk of hospital mortality in these patients.

## Materials and methods

### Study design and participants

We conducted a single-center, retrospective cohort study of patients admitted to all ICUs at the University of Kentucky Albert B. Chandler Hospital between August 2017 and July 2020. Inclusion criteria consisted of adult (age ≥18 years old) ICU patients that suffered from AKI requiring CRRT in whom clinicians prescribed NUF to achieve a mean daily patient fluid balance goal of negative ≥0.5 liters. The latter criterion restricted the cohort to only patients in whom the decision to prescribe NUF with CRRT (i.e., fluid removal) was determined by the clinicians at the bedside, therefore reducing indication bias.

**Fig 1:**
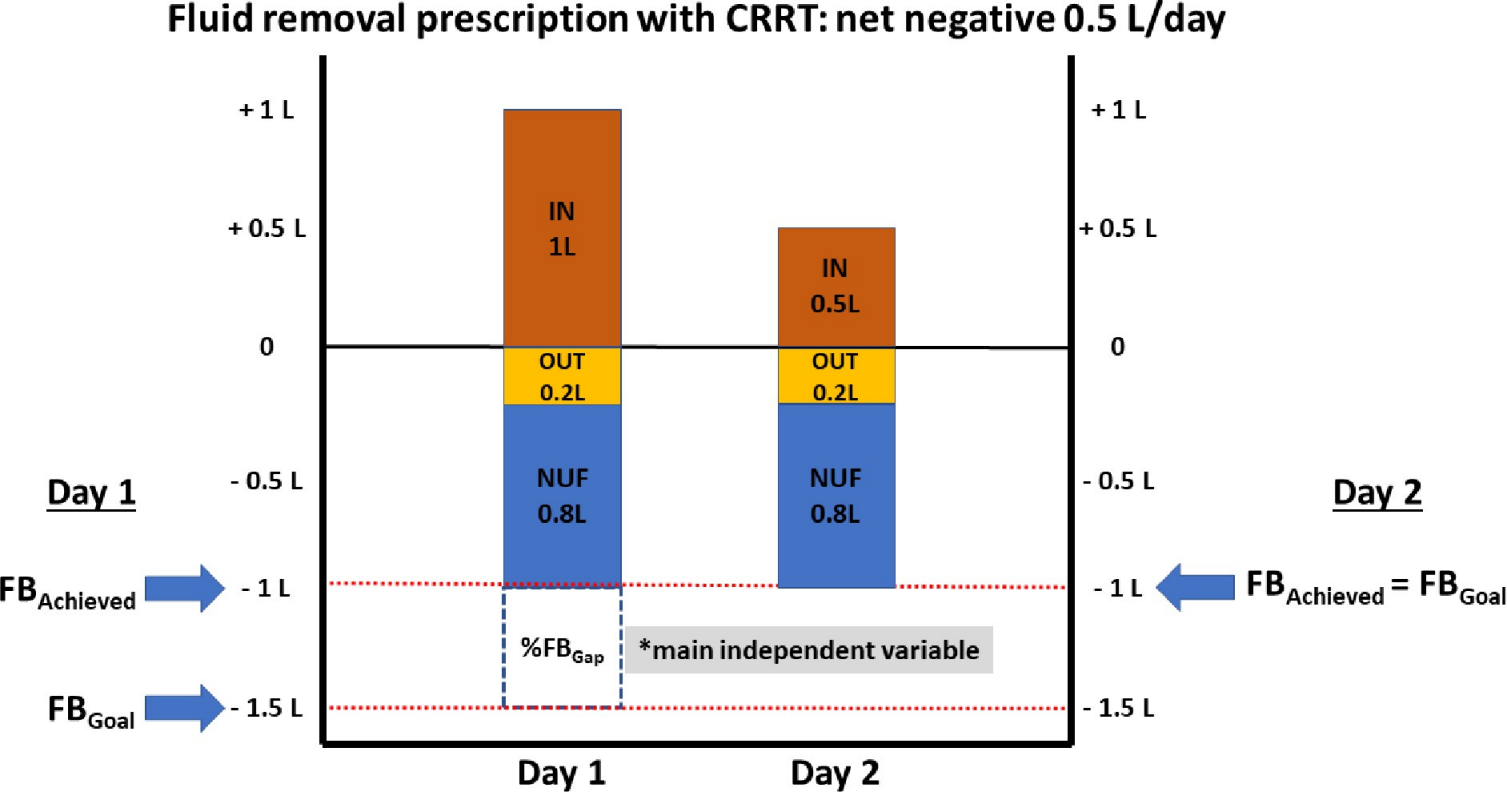
Conceptual map of fluid management parameters used during CRRT. In this patient example, the fluid removal prescription with CRRT is net negative 0.5 L per day for both represented days. One should note that despite similar NUF is attained in both days, fluid balance status is different in both days. On CRRT Day 1, FB_Achieved_ is 0 and therefore %FB_Gap_ is 100%. In contrast, on CRRT Day 2, FB_Achieved_ is -0.5 L and therefore %FB_Gap_ is 0%. Abbreviations: NUF (net ultrafiltration rate); FB_Goal_ (patient fluid balance goal); FB_Achieved_ (patient fluid balance achieved); %FB_Gap_ (Gap of patient fluid balance achieved vs. goal).

Patients were excluded if they had a diagnosis of end-stage kidney disease (ESKD) before index admission, had undergone kidney transplantation, or had more than 20% missing data related to daily achieved fluid balance, prescribed fluid balance, or NUF delivered with CRRT (**Fig 1**). To avoid interpreting implausible data likely related to documentation errors in electronic health records (EHR) flowsheets, we systematically excluded patients with fluid balance parameters above the 99.5 and below the 0.5 percentiles. If patients had more than one ICU stay requiring CRRT less than 6 hours apart within the same index hospitalization, it was considered a single ICU stay; otherwise it was considered separately. Notably, eligibility and timing of CRRT initiation and prescription of NUF according to patient fluid balance goals were determined by consultant nephrologists in collaboration with intensivists based on the institution’s standard of care practice. There were no changes in protocols or guidance for prescribing NUF throughout the study period. For detecting differences in %FB_Gap_ between patients who survived vs. died, a *post-hoc* power analysis revealed that a two-sample t-test would have at least 90% power to detect an effect size of d = 0.5 (0.979 was observed) with 235 patients per group at *α* = 0.01. The study was approved by the Institutional Review Board (IRB) from the University of Kentucky (17-0444-P1G). Given the observational nature of this investigation, informed consent was waived by the IRB.

### Data extraction

Data were gathered through automatic digital extraction from EHR and validated through comprehensive individual review of ~10% of EHRs. These data have been continuously collected, harmonized and validated as part of quality assurance procedures established at the University of Kentucky [15]. The presence of comorbidities was assessed using Charlson score [16], with individual comorbidities identified using ICD-9/10-CM codes. Baseline estimated glomerular filtration rate (eGFR) was determined using the CKD-EPI equation [17], utilizing the most recent outpatient (first option) or inpatient (second option) serum creatinine (sCr) value ≥7 days before hospital admission, but not more than 1 year prior to admission, or the sCr value at admission if no other value was available. Data related to patient fluid balance and CRRT parameters were extracted from specific ICU electronic flowsheets. Critical illness severity was assessed by calculating the Sequential Organ Failure Assessment score [18], using clinical data within the first 24 hours of ICU admission and CRRT initiation. Other clinical data included demographics and other extracorporeal organ support such as mechanical ventilation, extracorporeal membrane oxygenation (ECMO), or mechanical circulatory support (e.g., ventricular assist device [VAD] or intra-aortic balloon pump [IABP]).

### Fluid management assessment

All fluid management data were recorded hourly in dedicated electronic flowsheets. FO was calculated in reference to the ICU admission body weight using the percent FO formula proposed by Goldstein et al, [19] FO = Σ (fluid intake–fluid output *in liters*) / (weight *in kilograms*) × 100%. FO% was estimated at the time of CRRT initiation using fluid balance data from ICU admission to 1 hour prior to CRRT start. Fluid management during CRRT was evaluated according to 4 parameters:

1. *Net Ultrafiltration Rate or NUF (ml/kg/h)*, which corresponds to the net fluid removal achieved by the CRRT machine, expressed in milliliters per hour and adjusted per patient ICU admission weight
2. *Patient fluid balance goal or FB*_*Goal*_ *(ml/kg/h)*, which corresponds to the desired fluid balance goal determined by the clinician(s) at the bedside, expressed in milliliters per hour and adjusted per patient ICU admission weight
3. *Patient fluid balance achieved or FB*_*Achieved*_ *(ml/kg/h)*, which corresponds to the fluid balance achieved during CRRT, expressed in milliliters per hour and adjusted per patient ICU admission weight
4. *Gap of patient fluid balance achieved vs*. *goal or FB*_*Gap*_ *(%)*, which corresponds to the % gap of patient fluid balance achieved from the goal throughout the course of CRRT, expressed in percentage and calculated as follows:

$$\%FB\;Gap=\frac{FB\;Goal-FB\;Achieved}{FB\;Goal}\times 100\%$$

The fluid management parameters were adjusted by patient ICU admission body weight (*kilograms*) and the total time on CRRT (*hours*) to accommodate for patient size and the dynamic and heterogeneous nature of fluid management during CRRT. A conceptual map of these parameters is presented in **Fig 1**. For visualization purposes, patients were also classified in 3 clinical subgroups according to fluid balance status: 1) underachievers of FB_*Goal*_ (below the fluid balance goal) with overall positive fluid balance; 2) underachievers of FB_*Goal*_ (below the fluid balance goal) with overall negative fluid balance; and 3) achievers of FB_Goal_ (at or above the fluid balance goal) with overall negative fluid balance.

### Study outcomes

The primary study outcome was in-hospital mortality. A secondary outcome was the need for RRT in the last 48 h before hospital discharge as a surrogate of kidney recovery, but adjusting by the competing risk of death as a composite outcome (death or RRT in the last 48 h of hospitalization).

### Statistical analysis

Categorical data are presented as frequency and percentages. Continuous data are summarized with their median and interquartile ranges with only age being summarized with mean and standard deviation. Comparisons of patient characteristics according to hospital mortality were done using the chi-square test for categorical independent variables, and a t-test or Mann Whitney U test for continuous variables as appropriate. Covariates considered in the multivariable models were based on univariate analysis and clinical rationale, and consisted of demographics (age, sex, race); baseline eGFR; comorbidity based on Charlson score; acuity of illness according to SOFA score at the time of CRRT initiation, need of mechanical ventilation, need of ECMO or mechanical circulatory support (e.g., VAD, IABP).

Multivariable regression models were built using the Least Absolute Shrinkage and Selection Operator (LASSO) regression [20]. Models that had the smallest Extended Regularized Information Criterion (ERIC) [21] were selected as the final base models. Once these models were selected, they were redone with traditional logistic regression to obtain parameter estimates. Finally, if not already selected in the LASSO step, the main independent variables under investigation (%FB_Gap_ and NUF) were entered into the regression. This model building strategy produced our so called "final" model for interpretation and conclusions about the main independent variables under investigation. An exploratory investigation of the quadratic nature of %FB_Gap_ was also performed by including a quadratic term of %FB_Gap_ in the “final” regression model. The interactions between the two independent predictors and 1) CRRT duration and 2) the primary outcome were also examined. Restricted cubic splines were constructed to represent the adjusted relationship of the two main independent variables (%FB_Gap_ and NUF) and the primary study outcome (hospital mortality). Values below 2.5 and above 97.5 percentiles were excluded from the cubic splines to avoid implausible extrapolation of shapes of the relationship. R version 4.0, JMP®, Version *16* Pro [22] and SAS Institute Inc., Cary, NC were used for statistical analyses.

## Results

### Clinical characteristics

**Fig 2:**
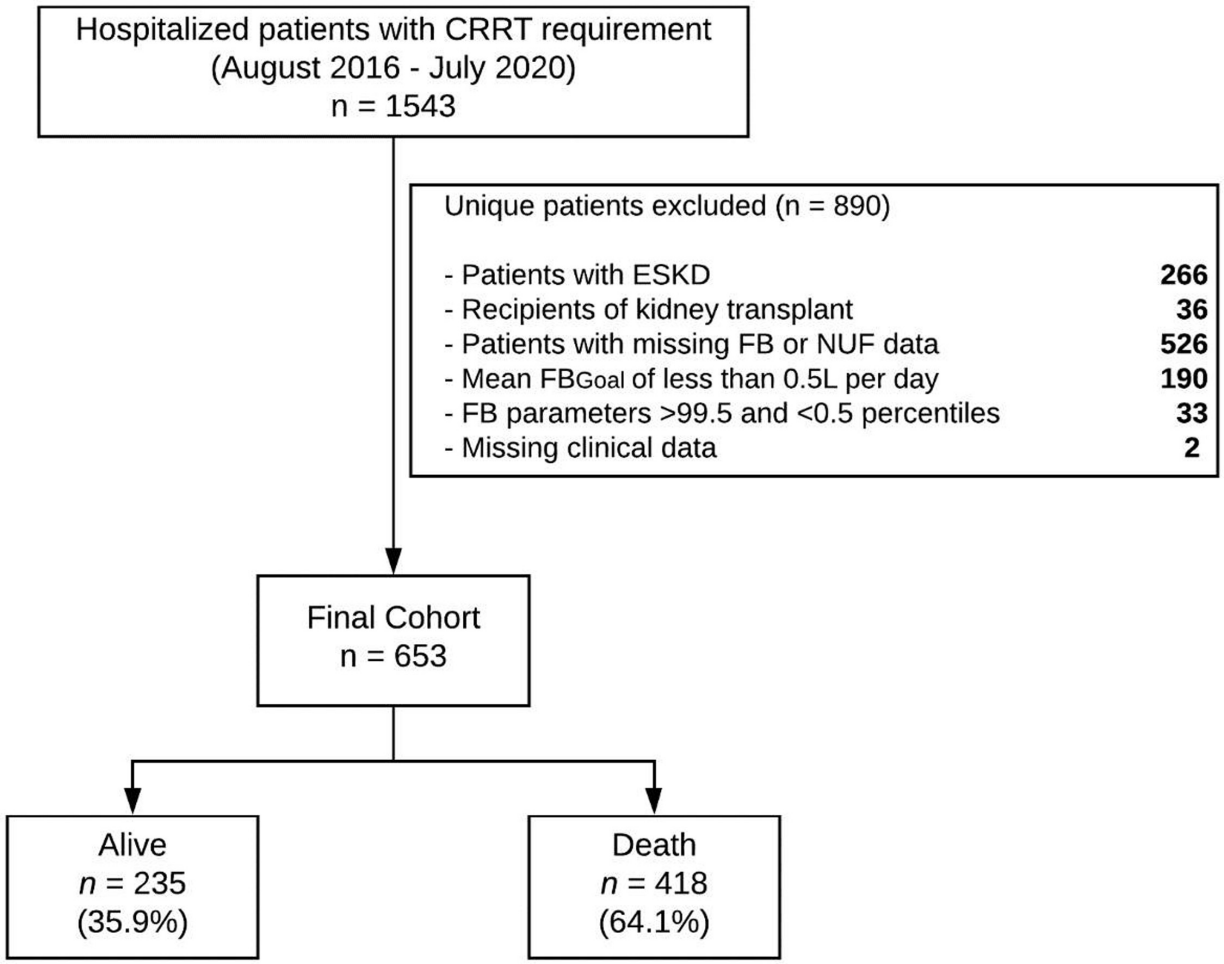
Cohort derivation. CRRT (continuous renal replacement therapy); ESKD (end-stage kidney disease); FB (fluid balance); FB_Goal_ (patient fluid balance goal); NUF (net ultrafiltration rate).

A total of 653 patients representing a median of 102.2 [55–205.8] patient-hours of CRRT were evaluated in the study. The cohort derivation algorithm with inclusion and exclusion criteria is detailed in **Fig 2**. A total of 235 of patients (36%) survived the hospitalization, while 418 (64%) died in the hospital. The mean (SD) age of the cohort was 56.7 (14.6) years, 61.9% were male and 91.3% white. Our cohort included 8 patients diagnosed with COVID-19 infection. As this was at the beginning of the pandemic, there were no established protocols related to processes of CRRT delivery specific to these patients.

Detailed clinical characteristics of the whole cohort and according to the hospital mortality outcome are presented in **Table 1**. Patients who died in the hospital were older (mean age 58.4 ± 14.1 vs. 53.7 ± 14.9 years, *p* <0.001) and had higher comorbidity based on Charlson scores. SOFA scores at the time of CRRT initiation were higher in patients that died than in those that survived (median 14 [12–16] vs. 12 [10–15], *p* <0.001). Patients that died received overall more ECMO/mechanical circulatory support than those that survived (**Table 1**).

**Table 1:** Patient characteristics in the whole cohort and according to hospital mortality.

	All cohort	Survived	Died	P-value
Number of patients	653	235	418	
**Demographics**				
Age, years	56.69 ± 14.55	53.65 ± 14.88	58.40 ± 14.09	<0.001
Male, n (%)	404 (61.87)	142 (60.43)	262 (62.68)	0.628
Race, n (%)				
• White	596 (91.27)	222 (94.47)	374 (89.47)	0.071
• Black	39 (5.97)	11 (4.68)	28 (6.70)	
• Other	18 (2.76)	2 (0.85)	16 (3.83)	
Weight, kg,	93.00 [77.70–109.92]	91.70 [77.70–109.10]	93.60 [77.58–110.20]	0.753
**Baseline kidney function**				
eGFR, ml/min/1.73m^2^	47.08 [23.06–77.08]	43.22 [20.09–75.31]	49.34 [23.90–80.29]	0.254
SCr, mg/dl	1.47 [0.99–2.63]	1.57 [1.04–2.92]	1.43 [0.96–2.52]	0.144
**Comorbidity**				
Charlson score	4.00 [2.00–7.00]	4.00 [2.00, 6.00]	4.00 [2.00, 7.00]	0.041
**RRT characteristics**				
CRRT days	4.26 [2.29–8.54]	5.00 [2.92–9.08]	3.71 [1.91–8.15]	<0.001
HD after CRRT, n (%)	223 (34.15)	170 (72.34)	53 (12.67)	<0.001
**Critical illness parameters**				
ICU LOS, days	10.00 [5.00–19.00]	14.00 [8.00–25.50]	8.00 [4.00–15.00]	<0.001
SOFA at ICU admission	12.00 [90.00–14.00]	11.00 [9.00–13.00]	12.00 [9.00–15.00]	0.004
SOFA at CRRT initiation	13.00 [11.00–16.00]	12.00 [10.00–15.00]	14.00 [12.00–16.00]	<0.001
Mechanical ventilation, n (%)	610 (93.42)	215 (91.49)	395 (94.50)	0.186
ECMO/mechanical circulatory support*, n (%)	104 (15.93)	25 (10.64)	79 (18.90)	0.008
**Fluid Balance During CRRT**				
%FO at CRRT start	2.81 [0.01–7.74]	3.16 [0.30–7.93]	2.56 [0.00–7.26]	0.350
Average Intake, L/day	2.97 [2.32–3.63]	2.72 [2.17–3.25]	3.17 [2.42–3.90]	<0.001
Average Output, L/day	3.08 [2.20–3.85]	3.29 [2.77–3.94]	2.93 [1.94–3.76]	<0.001
Average Net, L/day	-0.14 [-0.80 to 0.71]	-0.62 [-1.04 to -0.11]	0.17 [-0.54 to 1.17]	<0.001
Total Intake, L	13.36 [7.25–24.77]	13.75 [8.02–25.07]	13.14 [6.99–24.58]	0.130
Total Output, L	14.34 [7.06–27.68]	17.56 [9.92–29.45]	11.96 [4.93–25.62]	<0.001
Total Net, L	-0.95 [-5.00 to 2.39]	-3.46 [-7.45 to -0.48]	0.54 [-3.34 to 3.18]	<0.001
%FO per day of CRRT	-0.87 [-5.67 to 2.63]	-3.96 [-7.64 to -0.65]	0.61 [-3.54 to 3.81]	<0.001

*Use of ECMO, VAD or IABP. All continuous data are expressed as median [Q1-Q3] except age, which is expressed as mean (SD).

Abbreviations: CRRT (continuous renal replacement therapy); ECMO (extracorporeal membrane oxygenation); eGFR (estimated glomerular filtration); FO (fluid overload); HD (hemodialysis); IABP (intra-aortic balloon pump); ICU (intensive care unit); LOS (length of stay); SCr (serum creatinine); SOFA (sequential organ failure assessment score); VAD (ventricular assist device).

Median FO% estimated from ICU admission to CRRT start was 2.8% [0%-7.7%] in the whole cohort and did not differ among patients who died vs. survived (2.6% vs. 3.2%, *p* = 0.35). However, median FO% per day of CRRT was higher in patients that died vs. those that survived the hospitalization (0.61% [-3.54 to 3.81] vs. -3.96 [-7.64 to -0.65%], *p* <0.001, respectively) (**Table 1**).

### Fluid management during CRRT and hospital mortality

Although patient FB_Goal_ was similar in patients who died vs. in those that survived (median -0.75 [-1.20 to -0.49] vs. -0.80 [-1.18 to -0.52] ml/kg/h, *p* = 0.944, respectively), patients who survived achieved more negative fluid balance than non-survivors: FB_Achieved_ -0.25 [-0.52 to -0.05] vs. 0.06 [-0.26 to 0.62] ml/kg/h, *p* <0.001, respectively.

**Fig 3:**
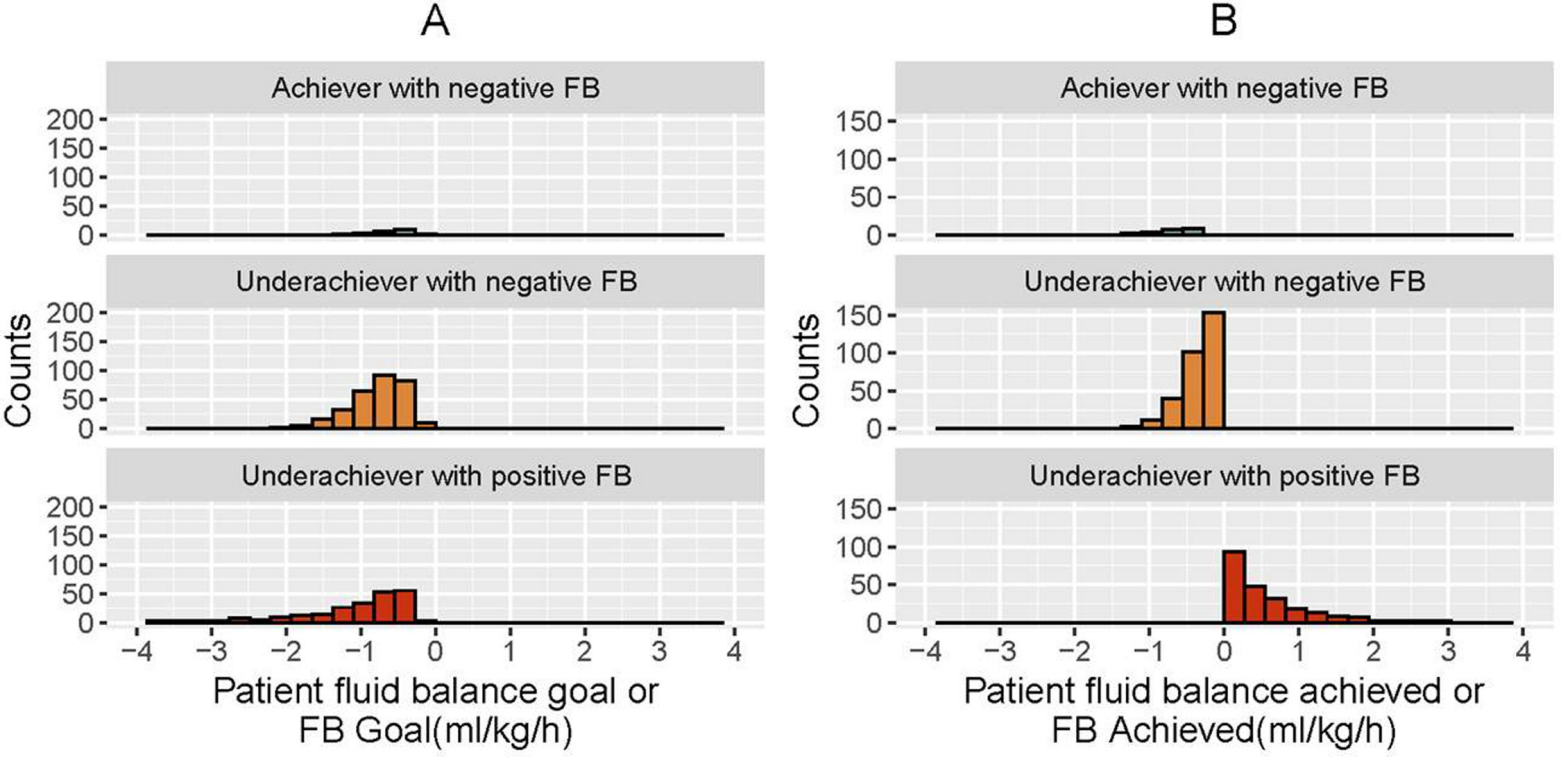
Graphic representation of patient FB_Goal_ (ml/kg/h, Panel A) and FB_Achieved_ (ml/kg/h, Panel B) classified in 3 clinical subgroups: 1) underachiever with positive fluid balance (red); 2) underachiever with negative fluid balance (orange); and 3) achiever with negative fluid balance (green). For visualization, data represented in the Figure exclude <2.5 and >97.5 percentiles.

**Fig 4:**
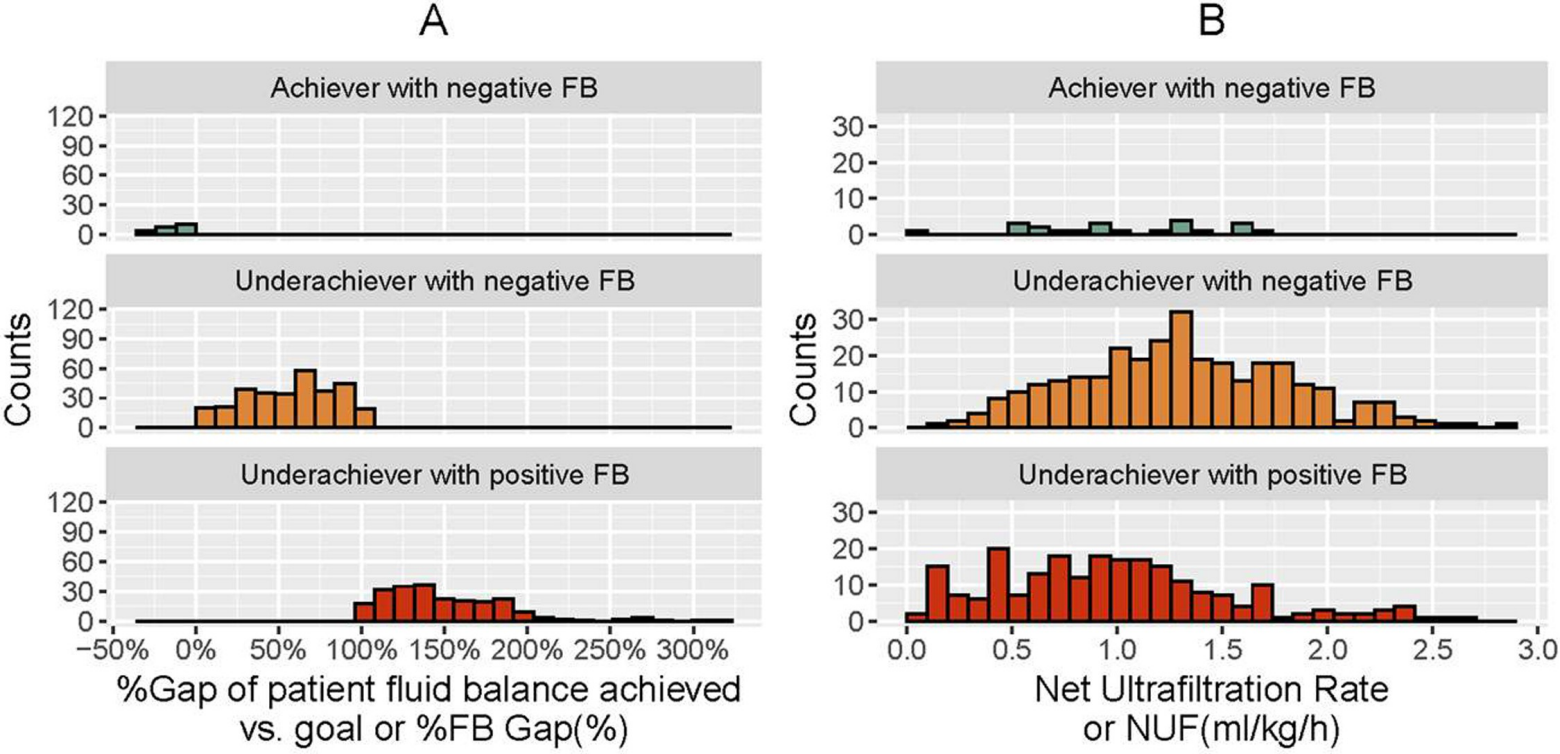
Graphic representation of %FB_Gap_ (Panel A) and NUF (ml/kg/h, Panel B) classified in 3 clinical subgroups: 1) underachiever with positive fluid balance (red); 2) underachiever with negative fluid balance (orange); and 3) achiever with negative fluid balance (green). Note that %FB_Gap_ better differentiate the 3 clinical subgroups than NUF. For visualization, data represented in the Figure exclude <2.5 and >97.5 percentiles.

**Fig 3** depicts the three clinical subgroups of patients according to fluid balance status: 1) underachievers of FB_Goal_ with positive fluid balance (mortality rate of 82.6%); 2) underachievers of FB_Goal_ with negative fluid balance (mortality rate of 50.5%); and 3) achievers of FB_Goal_ (mortality rate of 46.5%). This figure exhibits that FB_Achieved_ was frequently below the FB_Goal_ of negative fluid balance in most patients. This gap is represented as the %FB_Gap_ in **Fig 4A**. The distribution of %FB_Gap_ according to clinically relevant cutoffs (<20%, 20–50, >50%) is reported in **S1 Table**. In addition, **Fig 4B** depicts the NUF distribution in these clinical subgroups. An important observation is that NUF does not differentiate these three clinical subgroups given that the same NUF may represent a variety of patient fluid balance statuses.

NUF was lower in patients who died vs. survived: median 1.06 [0.63–1.47] vs. 1.22 [0.82–1.69] ml/kg/h, *p* <0.001, respectively. Consequently, %FB_Gap_ was higher in patients who died vs. survived: median 112.8% [61.5 to 165.7] vs. 64.2% [30.5 to 91.8], *p* <0.001, respectively (**Table 2**). There was no interaction between %FB_Gap_ * NUF and hospital mortality (*p* = 0.26). Similarly, for the outcome of hospital mortality, there was no interaction between %FB_Gap_ or NUF and the time on CRRT (*hours*) (*p* = 0.57 and 0.64, respectively). In multivariable models adjusted for relevant confounders, %FB_Gap_ but not NUF was independently associated with hospital mortality: adjusted OR (95% CI) 1.01 (1.01–1.02), *p* <0.001 for %FB_Gap_ and 0.96 (0.72–1.28), *p* = 0.771 for NUF (**Table 3**). Further, the quadric term of %FB_Gap_ was statistically significant (*p* <0.001) suggesting that the relationship of %FB_Gap_ with hospital mortality may be quadratic in nature rather than linear.

**Table 2:** CRRT parameters reflecting fluid management during CRRT in the whole cohort and according to study outcomes.

	**All cohort**	**Survived**	**Died**	**P-value**
Number of patients	653	235	418	
NUF, ml/kg/h	1.14 [0.69 to 1.58]	1.22 [0.82 to 1.69]	1.06 [0.63 to 1.47]	<0.001
FB_Goal_, ml/kg/h	-0.76 [-1.19 to -0.51]	-0.80 [-1.18 to -0.52]	-0.75 [-1.20 to -0.49]	0.944
FB_Achieved_, ml/kg/h	-0.07 [-0.40 to 0.32]	-0.25 [-0.52 to -0.05]	0.06 [-0.26 to 0.62]	<0.001
FB_Gap_, %	90.46 [46.26 to 141.69]	64.22 [30.49 to 91.83]	112.82 [61.46 to 165.67]	<0.001
		**Survived/No RRT in last 48h**	**Died/RRT in last 48h**	**P-value**
Number of patients		142	511	
NUF, ml/kg/h		1.20 [0.65 to 1.66]	1.12 [0.70 to 1.53]	0.240
FB_Goal_, ml/kg/h		-0.76 [-1.21 to -0.52]	-0.77 [-1.19 to -0.50]	0.845
FB_Achieved_, ml/kg/h		-0.23 [-0.47 to -0.02]	-0.01 [-0.34 to 0.46]	<0.001
FB_Gap_, %		63.81 [33.61 to 97.07]	98.42 [50.05 to 153.58]	<0.001

Abbreviations: NUF (net ultrafiltration rate); FB_Goal_ (patient fluid balance goal); FB_Achieved_ (patient fluid balance achieved); %FB_Gap_ (Gap of patient fluid balance achieved vs. goal).

**Table 3:** Multivariable logistic regression model of %FB_Gap_ and NUF as the independent variables and a) hospital mortality and b) death or RRT in the last 48 h of hospitalization as the dependent variables.

	A. Hospital Mortality		B. Death/RRT in last 48h	
Variables	OR (95% CI)	P-value	OR (95% CI)	P-value
Age, per 1 year	1.02 [1.01–1.04]	<0.001	1.02 [1.01–1.04]	0.002
SOFA at CRRT start, per 1 unit score	1.13 [1.07–1.20]	<0.001	1.10 [1.03–1.17]	0.003
ECMO/mechanical circulatory support*	2.35 [1.37–4.17]	0.003	2.97 [1.49–6.61]	0.005
%FB_Gap_, per 1% increase	1.01 [1.01–1.02]	<0.001	1.01 [1.00–1.01]	<0.001
NUF, per 1 ml/kg/h	0.96 [0.72–1.28]	0.771	1.26 [0.91–1.76]	0.176

*Use of ECMO, VAD or IABP.

Abbreviations: CRRT (continuous renal replacement therapy); ECMO (extracorporeal membrane oxygenation); %FB_Gap_ (Gap of patient fluid balance achieved vs. goal); IABP (intra-aortic balloon pump); NUF (net ultrafiltration rate); SOFA (sequential organ failure assessment score); VAD (ventricular assist device).

**Fig 5:**
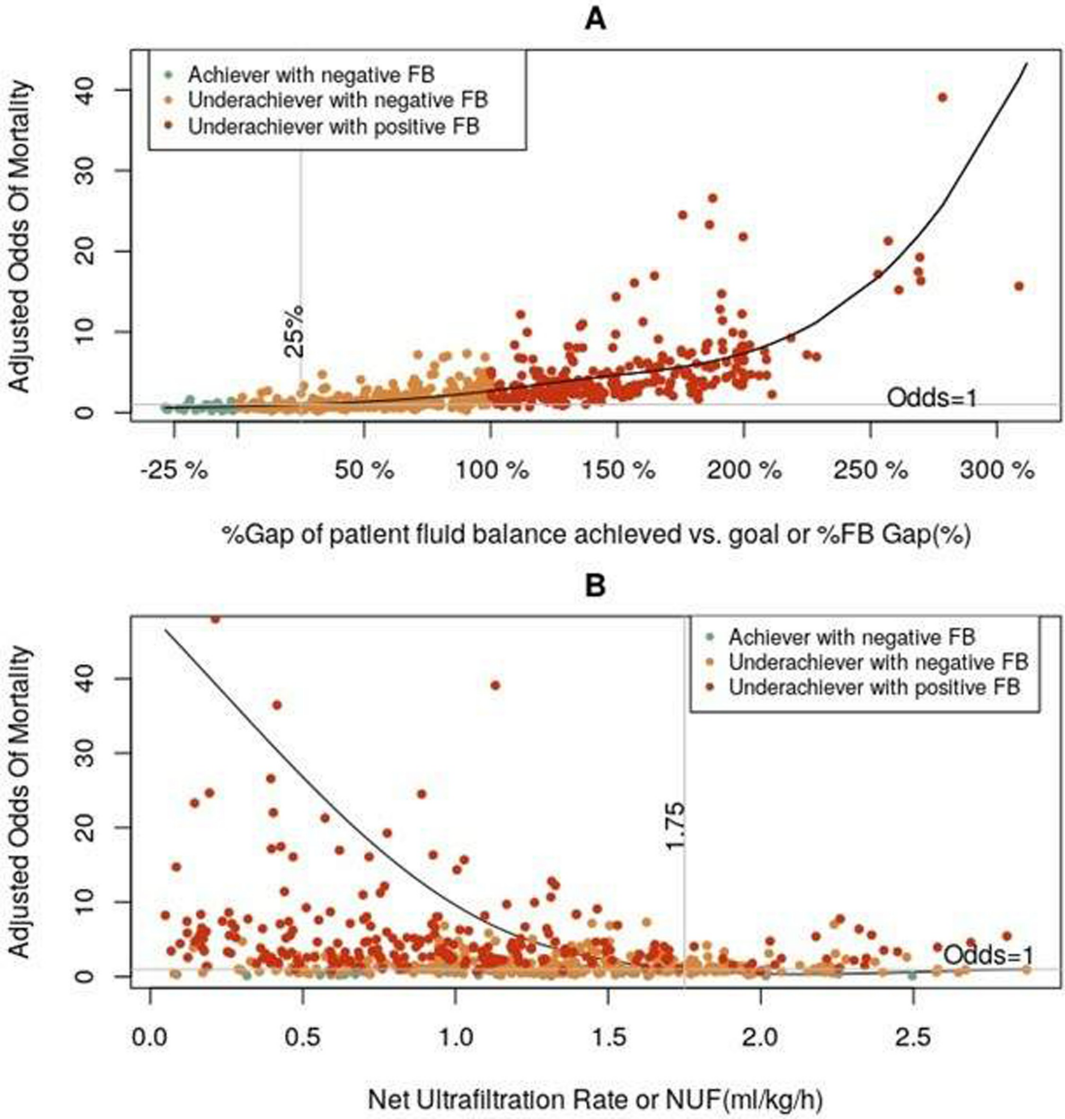
Restricted cubic splines representing the adjusted relationship of A) %FB_Gap_ and B) NUF (ml/kg/h) with hospital mortality according to final multivariable models. Note that %FB_Gap_ better differentiate the 3 clinical subgroups than NUF for the association with mortality.

**Fig 6:**
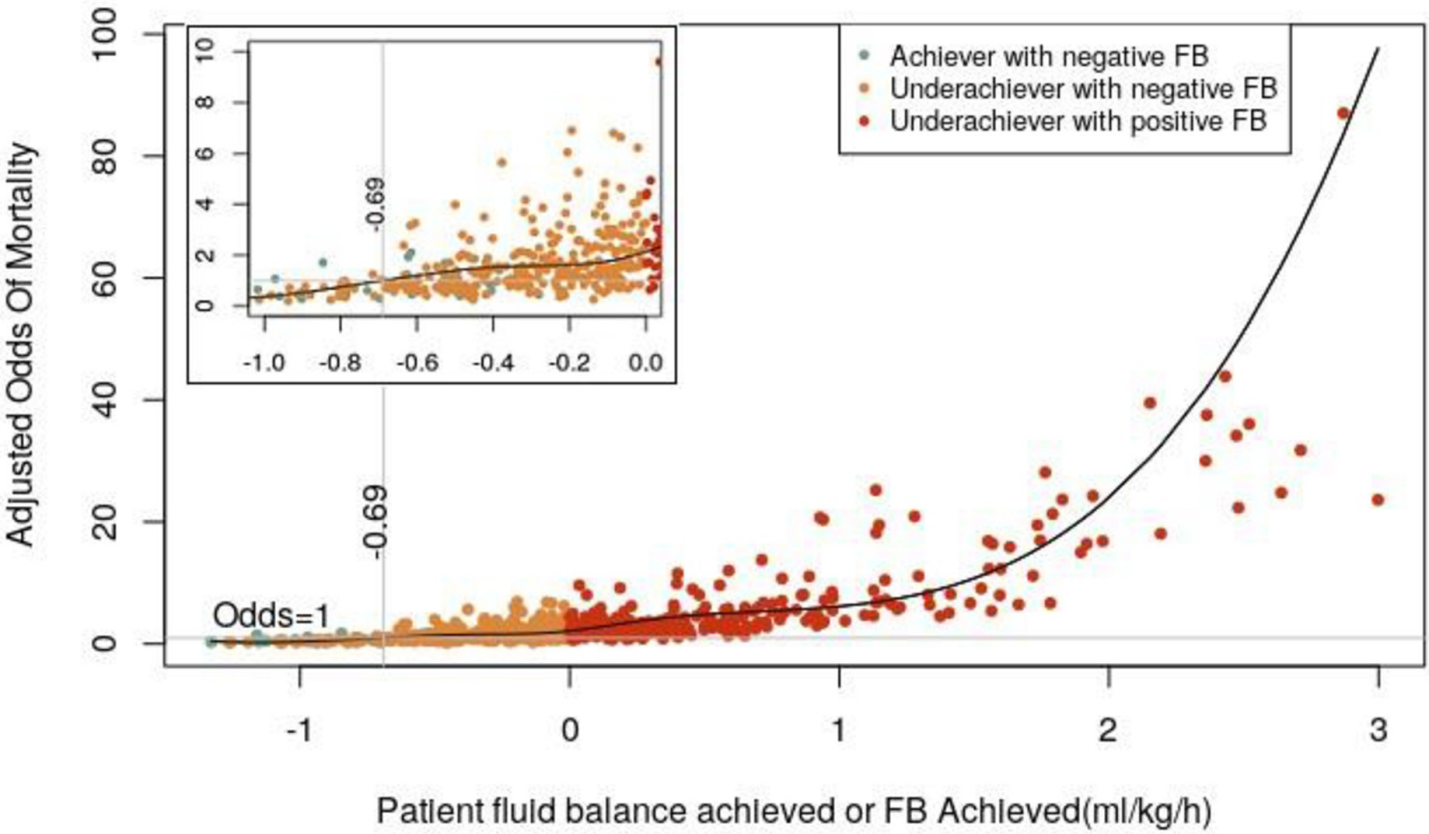
Restricted cubic splines representing the adjusted relationship of FB_Achieved_ (ml/kg/h) with hospital mortality according to final multivariable models. Note that mortality in some patients with negative FB may be influenced by other factors beyond FB_Achieved_ and covariates in the model as depicted by the zoomed portion of the Figure.

**Fig 5** depicts the adjusted relationships between these two parameters, %FB_Gap_ (**Fig 5A**) and NUF (**Fig 5B**), and hospital mortality. Finally, the adjusted relationship between FB_Achieved_ and hospital mortality is represented in **Fig 6**. These figures collectively suggest that higher %FB_Gap_, lower NUF and less negative FB_Achieved_ associate with increased risk of hospital mortality in this study population. Other clinical parameters associated with hospital mortality were older age, higher SOFA score at CRRT start, and the need of ECMO or mechanical circulatory support (**Table 3**).

### Fluid management during CRRT and kidney recovery

Although patient FB_Goal_ and NUF were similar in patients who recovered *vs*. did not recover kidney function by hospital discharge, FB_Achieved_ was less negative in patients who died or were still receiving RRT in the last 48 h of hospitalization vs. in those who survived and were liberated of RRT: median -0.01 [-0.34 to 0.46] vs. -0.23 [-0.47 to -0.02] ml/kg/h, *p* <0.001, respectively. Consequently, %FB_Gap_ was higher in patients who died or were still receiving RRT in the last 48 h of hospitalization vs. survivors who were liberated of RRT: median 98.4% [50.1 to 153.6] vs. 63.8% [33.6 to 97.1], *p* <0.001, respectively (**Table 2**). However, these differences dissipated when only survivors were evaluated (**S2 Table**).

In multivariable models, %FB_Gap_ but not NUF was independently associated with death or RRT in the last 48 h of hospitalization: adjusted OR (95% CI) 1.01 (1.00–1.01), *p* <0.001 for %FB_Gap_ and 1.26 (0.91–1.76), *p* = 0.176 for NUF (**Table 3**). However, the significant association of %FB_Gap_ with death or RRT in the last 48 h of hospitalization dissipated when only survivors were evaluated (**S2 Table**). In survivors, NUF was independently associated with RRT in the last 48 h of hospitalization: adjusted OR 1.55 (1.03–2.38), *p* = 0.039 (**S3 Table**).

## Discussion

The main finding of this study is that in critically ill patients with AKI requiring CRRT in whom clinicians prescribed negative fluid balance, the %FB_Gap_ (% gap between prescribed vs. achieved patient fluid balance during CRRT) was independently associated with increased risk of hospital mortality. This study also highlights that underachievement of patient fluid balance goals during CRRT is very frequent in clinical practice, and therefore incorporating clinician prescription of fluid balance during CRRT provides valuable information, in addition to NUF, for risk-classification of adverse outcomes in these patients. This study is relevant because the examination of the association between %FB_Gap_ and mortality in a group of patients in whom clinicians prescribed average negative fluid balance during CRRT limits indication bias inherent to the evaluation of NUF without inclusion of the patient fluid balance goal prescribed by clinicians. The latter makes our study findings more generalizable and reproducible in the subset of critically ill patients in whom fluid removal is a key treatment goal of CRRT, while providing critical data to validate %FB_Gap_ as a quality indicator of CRRT delivery. Importantly, identification of specific causes of this %FB_Gap_ and interventions to narrow this gap through better patient fluid status evaluation and/or NUF delivery are critical areas of investigation by our group and others.

Similar to the % gap between prescribed vs. achieved total effluent dose of CRRT [23], %FB_Gap_ is an important CRRT quality indicator that should be systematically and dynamically monitored throughout the delivery of CRRT [24]. For example, a recent report by the Acute Disease Quality Initiative group recommended that %FB_Gap_ should be less than 20% on average. However, the median %FB_Gap_ in our cohort was 90.5% with only 8.7% of patients reaching the target of less than 20%. This clearly highlights the need for interventions to close this gap. In day to day practice, fluid management during CRRT is prescribed per specific patient fluid balance goals within a predefined period of time [25]. If negative fluid balance to manage FO in a patient on CRRT is desired, clinicians prescribe -for example- patient fluid balance as net negative FB of 100 ml/h to target net negative FB of ~2.4 liters in the next 24 h. To achieve this goal, bedside ICU or dialysis nurses calculate specific NUF targets each hour and program the CRRT machine to execute this hourly NUF. This calculation takes into consideration the patient fluid balance in the prior hour plus carried deficits from prior hour(s) to achieve the desired negative fluid balance goal at the end of the predefined period of time. This is a continuum of hourly-basis practice that requires clear communication between nurses and clinicians as well as logistics for monitoring. Therefore, NUF is a dynamic metric that is clearly linked not only to patient fluid balance but also to the clinicians prescription of fluid management during CRRT. To develop the fluid management parameters utilized in this study, we used hourly data collected in electronic flowsheets that were properly validated through an established quality assurance program [15].

The inability to achieve the desired patient negative fluid balance generates a %FB_Gap_ that can worsen over time if the causes generating the gap are not identified. The cause(s) could be multifactorial and it may be related to the inability to effectively execute NUF at the bedside (e.g., interrupted CRRT due to access issues, machine malfunction, circuit clotting or impaired logistics at the corresponding institution, among others). A worsening %FB_Gap_ could also be related to patient intolerance to NUF due to underlying hypotension/acute illness processes and sometimes due to inadequate assessment (e.g., too high NUF intensity) by the clinician. These observations have been highlighted in two recent multinational surveys that revealed significant practice variations in regard to fluid management during CRRT [26, 27]. It is possible that further validation of %FB_Gap_ as a quality indicator of CRRT and better recognition of the impact of %FB_Gap_ on clinical outcomes may raise awareness for its routine monitoring and promote strategies to implement more effective delivery of NUF in critically ill patients with FO receiving CRRT. Further, the evolving utilization of point-of-care ultrasonography for volume status and cardiac function assessment in the ICU could assist clinicians to better tailor patient fluid balance goals during CRRT [28].

Prior observational studies have evaluated the association between NUF and mortality. A report by Murugan et al studied a cohort of patients with FO ≥5% prior to CRRT initiation and showed that NUF intensity >25 ml/kg/day (vs. ≤ 20 ml/kg/day) was associated with lower 1-year risk-adjusted mortality [12]. Similar to our findings, this study showed that higher NUF associated with lower mortality in patients with a clinical diagnosis of FO (**Fig 5B**). However, this study did not evaluate clinician fluid balance prescription (e.g., FB_Goal_ and %FB_Gap_) as in our study. In a subsequent study, Murugan et al conducted a post-hoc analysis of the Randomized Evaluation of Normal vs. Augmented Level (RENAL) of Renal Replacement Therapy trial and showed that NUF rates greater than 1.75 ml/kg/h (highest tertile) vs. less than 1.01 ml/kg/h (lowest tertile) were associated with lower survival [13]. Different to our study, this study did not restrict the cohort to patients with FO at the time of CRRT initiation or in whom clinicians prescribed NUF to target negative fluid balance. Subsequent studies have evaluated NUF restricted to the first 48 h of CRRT and have showed a similar association with mortality outcomes (NUF rates >1.75mL/kg/h vs. <1.01mL/kg/h were associated with increased mortality) [29]. Another study attempted to address indication bias by evaluating interactions of NUF with possible mediators such as patient fluid balance (achieved but not goal), hemodynamic status and electrolyte disturbances. This study showed that NUF >1.75 ml/kg/h was independently associated with increased hospital mortality, and that this effect appeared not mediated by the aforementioned factors [30]. One should note that FB_Goal_ or %FB_Gap_ was not evaluated in any of these studies.

A more recent report concluded that the relationship between NUF and mortality may be partially influenced by the ability of the patient to achieve negative daily fluid balance, particularly in patients with FO at the beginning of CRRT [14]. Similarly, another study found that in patients with AKI on CRRT a decrease in cumulative FB was independently associated with lower mortality. Importantly, this study only included 89 patients with FO >10% at CRRT initiation [31]. These findings highlight -concordant with our study- that NUF should be interpreted in the context of the patient fluid balance status. Taken together, observational evidence is highly supportive of personalized approaches to achieve desired negative fluid balance in patients with FO requiring CRRT because the impact of FO may vary between patients and “high intensity” NUF for one patient may be “low intensity” for a different patient. In this context, clinical trials should target interventions to mitigate FO by optimizing assessment and delivery of NUF during CRRT, the latter with safety boundaries as “high intensity” NUF may predispose patients to hemodynamic instability, myocardial stunning and other complications [32, 33]. Certainly, precision in the evaluation of FO is mandatory for implementation of these trials. %FB_Gap_ could serve -in this context- to evaluate adherence to the intervention protocol and guide adjustments in FB_Goal_ and/or NUF in a dynamic fashion throughout a clinical trial.

Our study has notable strengths. First, to our knowledge, this is the first study that utilizes clinician fluid balance prescription during CRRT (FB_Goal_) to calculate the %FB_Gap_. Second, we showed the importance of %FB_Gap_ as a quality indicator of CRRT delivery given its independent association with hospital mortality in robust multivariable models. Further, %FB_Gap_ may constitute a feasible operational metric to utilize in pragmatic clinical trials. Third, we addressed indication bias with a different approach than prior observational studies by restricting the study cohort to patients in whom clinicians prescribed NUF to achieve average negative fluid balance during CRRT.

Some limitations of our study are also worth noting. First, our study is single-center and observational in nature and therefore cannot infer causality in the relationship between %FB_Gap_ and hospital mortality. Second, the causes driving the %FB_Gap_ are likely multifactorial and unable to be determined by this study. Therefore, residual unmeasured confounding influencing the association between %FB_Gap_ and hospital mortality is possible. Nonetheless, we comprehensively evaluated our study hypothesis in multivariable models to limit this possibility. Studies evaluating interventions to guide fluid regulation with CRRT (e.g., protocolized NUF, dynamic assessment of patient tolerance to fluid removal, etc.) are needed to better differentiate patient intrinsic factors vs. CRRT processes as the main drivers of the observed %FB_Gap_. Third, our study design limits indication bias which is an unresolved problem in prior observational studies but introduces selection bias, and therefore our results could only apply to critically ill patients with AKI and prescription of negative fluid balance during CRRT. Fourth, our study adjusted fluid management parameters according to the patient ICU admission body weight, which is a metric that could be influenced by the pre-ICU clinical course and is not always available through electronic bed scales in resource-limited hospital settings. Fifth, more than 90% of the study cohort is white and therefore additional studies representing other racial/ethnic groups are needed.

In summary, we evaluated a novel CRRT parameter derived from the gap between prescribed vs. achieved patient fluid balance during CRRT (%FB_Gap_). We found an independent association between higher %FB_Gap_ and increased risk of hospital mortality in critically ill patients with AKI on CRRT in whom clinicians prescribed NUF to achieve negative fluid balance during CRRT. Given that FO in patients on CRRT constitutes a potentially modifiable risk factor that may impact mortality outcomes, operationalizing fluid management interventions during CRRT with a low %FB_Gap_ by dynamically adjusting FB_Goal_ and NUF may be a novel precision medicine strategy that could be tested in clinical trials. Better tools to more accurately and dynamically assess FO and patient FB_Goal_ are needed to guide fluid management via CRRT. Similar to CRRT prescribed vs. delivered dose, %FB_Gap_ represents a novel quality indicator of CRRT delivery that requires more systematic evaluation and widespread implementation.

## Supporting information

S1 Table Distribution of %FB_Gap_ in the three clinical subgroups. Abbreviations: %FB_Gap_ (Gap of patient fluid balance achieved vs. goal); FB = fluid balance.
(DOCX)

S2 Table CRRT parameters reflecting fluid management during CRRT in survivors only and according to kidney recovery status determined as RRT in the last 48 h of hospitalization. Abbreviations: NUF (net ultrafiltration rate); FB_Goal_ (patient fluid balance goal); FB_Achieved_ (patient fluid balance achieved); %FB_Gap_ (Gap of patient fluid balance achieved vs. goal).
(DOCX)

S3 Table Multivariable logistic regression model of %FB_Gap_ and NUF as the independent variables and RRT in the last 48 h of hospitalization (surrogate of kidney recovery) as the dependent variable in survivors only. *Use of ECMO, VAD or IABP. Abbreviations: CRRT (continuous renal replacement therapy); ECMO (extracorporeal membrane oxygenation); %FB_Gap_ (Gap of patient fluid balance achieved vs. goal); IABP (intra-aortic balloon pump); NUF (net ultrafiltration rate); SOFA (sequential organ failure assessment score); VAD (ventricular assist device).
(DOCX)
