# Supplementary material for: Assessment of prescribed vs. achieved fluid balance during continuous renal replacement therapy and mortality outcome
Source: PLoS One. 2022 Aug 25;17(8):e0272913. doi: 10.1371/journal.pone.0272913 (PMC9409548; doi:10.1371/journal.pone.0272913)
Supplement: S1 Table — Abbreviations: %FBGap (Gap of patient fluid balance achieved vs. goal); FB = fluid balance. (DOCX) [file pone.0272913.s001.docx]

**Table S1:** Distribution of %FB_Gap_ in the three clinical subgroups

|  | **Achiever with negative FB** | **Underachiever with negative FB** | **Underachiever with positive FB** |
| --- | --- | --- | --- |
| Number of patients | 43 | 329 | 281 |
| **%FB_Gap_ above goal** | | | |
| <20% | 17 (39.6%) | -- | -- |
| 20-50% | 13 (30.2%) | -- | -- |
| >50% | 13 (30.2%) | -- | -- |
| **%FB_Gap_ below goal** | | | |
| <20% | -- | 40 (12.2%) | 0 (0%) |
| 20-50% | -- | 94 (28.5%) | 0 (0%) |
| >50% | -- | 195 (59.3%) | 281 (100%) |

*Abbreviations: %FB_Gap_ (Gap of patient fluid balance achieved vs. goal); FB =fluid balance.*
