# Supplementary material for: Assessment of prescribed vs. achieved fluid balance during continuous renal replacement therapy and mortality outcome
Source: PLoS One. 2022 Aug 25;17(8):e0272913. doi: 10.1371/journal.pone.0272913 (PMC9409548; doi:10.1371/journal.pone.0272913)
Supplement: S2 Table — Abbreviations: NUF (net ultrafiltration rate); FBGoal (patient fluid balance goal); FBAchieved (patient fluid balance achieved); %FBGap (Gap of patient fluid balance achieved vs. goal). (DOCX) [file pone.0272913.s002.docx]

**Table S2:** CRRT parameters reflecting fluid management during CRRT in survivors only and according to kidney recovery status determined as RRT in the last 48 h of hospitalization

|  | **Survivors only** | **No RRT in last 48h** | **RRT in last 48h** | **P-value** |
| --- | --- | --- | --- | --- |
| Number of patients | 235 | 142 | 93 |  |
| NUF, ml/kg/h | 1.22 [0.82-1.69] | 1.20 [0.65-1.66] | 1.30 [0.94-1.73] | 0.089 |
| FB_Goal_, ml/kg/h | -0.80 [-1.18 to -0.52] | -0.76 [-1.21 to -0.52] | -0.82 [-1.05 to -0.52] | 0.746 |
| FB_Achieved_, ml/kg/h | -0.25 [-0.52 to -0.05] | -0.23 [-0.47 to -0.02] | -0.28 [-0.60 to -0.09] | 0.150 |
| FB_Gap_, % | 64.22 [30.49-91.83] | 63.81 [33.61-97.07] | 64.22 [28.76-87.56] | 0.527 |

*Abbreviations: NUF (net ultrafiltration rate); FB_Goal_ (patient fluid balance goal); FB_Achieved_ (patient fluid balance achieved); %FB_Gap_ (Gap of patient fluid balance achieved vs. goal).*
