# Supplementary material for: Assessment of prescribed vs. achieved fluid balance during continuous renal replacement therapy and mortality outcome
Source: PLoS One. 2022 Aug 25;17(8):e0272913. doi: 10.1371/journal.pone.0272913 (PMC9409548; doi:10.1371/journal.pone.0272913)
Supplement: S3 Table — *Use of ECMO, VAD or IABP. Abbreviations: CRRT (continuous renal replacement therapy); ECMO (extracorporeal membrane oxygenation); %FBGap (Gap of patient fluid balance achieved vs. goal); IABP (intra-aortic balloon pump); NUF (net ultrafiltration rate); SOFA (sequential organ failure assessment score); VAD (ventricular assist device). (DOCX) [file pone.0272913.s003.docx]

**Table S3:** Multivariable logistic regression model of %FB_Gap_ and NUF as the independent variables and RRT in the last 48 h of hospitalization (surrogate of kidney recovery) as the dependent variable in survivors only

|  | **RRT in the last 48h** | | | | | | |
| --- | --- | --- | --- | --- | --- | --- | --- |
| **Variables** | | **OR (95% CI)** | | **P-value** | |  |  |
| Age, per 1 year | | | 1.01 [0.99-1.03] | | 0.233 | |  |
| SOFA at CRRT start, per 1 unit score | | | 1.01 [0.93-1.10] | | 0.794 | |  |
| ECMO/mechanical circulatory support* | | | 2.20 [0.87-5.72] | | 0.098 | |  |
| %FB_Gap_, per 1% increase | | | 1.00 [0.99-1.00] | | 0.853 | |  |
| NUF, per 1 ml/kg/h | | | 1.55 [1.03-2.38] | | 0.039 | |  |

*Use of ECMO, VAD or IABP.

*Abbreviations: CRRT (continuous renal replacement therapy); ECMO (extracorporeal membrane oxygenation); %FB_Gap_ (Gap of patient fluid balance achieved vs. goal); IABP (intra-aortic balloon pump); NUF (net ultrafiltration rate); SOFA (sequential organ failure assessment score); VAD (ventricular assist device).*
